# Single-Site Catalyst for the Synthesis of Disentangled Ultra-High-Molecular-Weight Polyethylene

**DOI:** 10.3390/polym17010095

**Published:** 2025-01-01

**Authors:** Jian Chen, Shuzhang Qu, Xinwei Li, Yiming Wei, Qian Li, Zhao Wen, Zifang Guo

**Affiliations:** Sinopec Key Laboratory of Research and Application of Medical and Hygienic Materials, SINOPEC (Beijing) Research Institute of Chemical Industry Co., Ltd., No. 14 Beisanhuan Donglu, Chao Yang District, Beijing 100013, China; qushzh.bjhy@sinopec.com (S.Q.); lixw.bjhy@sinopec.com (X.L.); weiym.bjhy@sinopec.com (Y.W.); liq.bjhy@sinopec.com (Q.L.); wenzh.bjhy@sinopec.com (Z.W.)

**Keywords:** disentangled UHMWPE, entanglement, single-site catalyst, solid-state processing

## Abstract

Disentangled ultra-high-molecular-weight polyethylene (*d*-UHMWPE) solves the problem of the difficult processing of traditional UHMWPE caused by entanglements between molecular chains. In this review, we look into the innovative realm of nascent disentangled UHMWPE, concentrating on the recent advances achieved through the in situ polymerization of ethylene by single-site catalysts. The effect of single-site catalysts and polymerization conditions on the molecular characteristics is discussed in detail from the perspective of mechanism and DFT calculations. The key factors to low entanglement are revealed, which have instructive implications for the development of new single-site catalytic systems that can generate *d*-UHMWPE more efficiently and become closer to industrial production. The progress in the preparation for nascent *d*-UHMWPE with homogeneous and heterogeneous single-site catalysts is systematically reviewed. Rheology and DSC can be used to characterize the degree of entanglement. High-modulus and high-strength biaxial films, tapes, and fibers are obtained by the solid-state processing of these nascent *d*-UHMWPE.

## 1. Introduction

**Figure 1:**
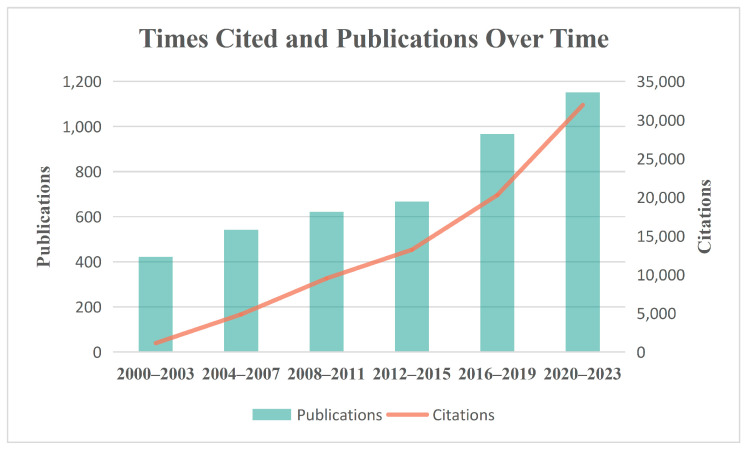
Statistics of the publication and citation of articles related to low-entanglement UHMWPE since 2000. Source: Web of Science (Keywords: “UHMWPE” or “Ultra-high-molecular-weight polyethylene” and “disentangled”).

Ultra-high-molecular-weight polyethylene (UHMWPE) is an essential engineering plastic with excellent properties, such as high impact resistance, self-lubrication, weak adhesion, good corrosion stability, and biocompatibility [1]. In particular, its high toughness, high abrasive resistance, low coefficient of friction, chemical resistance, and low density make it an ideal material for lightweight engineering [2] as well as medical equipment [3,4,5,6] applications. Commercially available UHMWPE is mainly prepared by heterogeneous Ziegler−Natta (Z−N) catalytic systems, which is mainly composed of titanium chloride (TiCl_4_) supported by magnesium chloride (MgCl_2_). The active species are randomly distributed on the surface of the carrier, and their proximity to one another makes it easy for the growth chains to become entangled. Furthermore, the polymerization temperature used in the industrial production is usually high, generally greater than 60 °C, so that the chain grows considerably more quickly than the rate of crystallization, which in consequence drives the formation of entanglement [4,7]. A high degree of entanglement in the polymer chain will restrict the molecular chain’s orientation, diffusion, and relaxation behavior [8]. This will cause a significant rise in the melt viscosity and challenging processing issues that cannot be resolved by traditional melt processing techniques like injection molding or blow molding. Also, the processing of the entangled UHMWPE will cause grain boundaries or fusion defects. Its mechanical properties are only 1/3 of the theoretical value because of chain entanglement [9]. Recent progresses demonstrated significant improvements in the processing and mechanical properties of *d*-UHMWPE compared to the traditional UHMWPE. For instance, *d*-UHMWPE has a stronger impact resistance because of less morphological defects [10]. Moreover, the products of *d*-UHMWPE exhibit remarkable enhancements in yield strength, tensile strength, and tensile modulus [11,12]. *d*-UHMWPE not only manifested an outstanding mechanical performance but also exhibited a remarkable thermal conductivity through uniaxial and biaxial stretching [13]. It was proposed that heat conduction in *d*-UHMWPE films results from longitudinal atomic vibrations that are ballistic within the extended crystal phase and are scattered primarily by reflections at the boundaries between the crystals [14]. Additionally, its low entanglement characteristic can improve UHMWPE’s compatibility with the mix as well as its impact on the blend’s toughening and reinforcing. The retarded thermal motion response caused by the entanglements between chains in traditional UHMWPE will lead to restricted compatibility with other polymers. It was discovered that *d*-UHMWPE is more compatible with high-density polyethylene (HDPE) and can enhance HDPE’s mechanical performance when compared to commercial entangled UHMWPE [15]. Over the past 20 years, research on UHMWPE and low entanglement has rapidly become a hotspot in the realm of materials science [16,17,18,19]. The statistical data in Figure 1 show a sharp increase in the quantity of relevant publications and citations. For UHMWPE, achieving minimal entanglement is a crucial issue that needs to be resolved.

Currently, gel spinning technology applied at the processing stage is the primary means for achieving low entanglement [20,21]. In this method, UHMWPE is dissolved in an appropriate solvent to obtain a diluted solution, which reduces the entanglement between the polymerization chains. The spinning process is then carried out through the spinneret. There are other concerns with this method as well. For example, the material must be swollen first using plenty of specific solvents, and the residue of the solvent after spinning affects the performance of the processed product. In addition to reducing the entanglement degree during processing, more research at this stage is conducted to directly generate UHMWPE with a low-entanglement density in situ from polymerization, generally known as nascent *d*-UHMWPE. There are two major methods to reduce the entanglement density in the polymerization stage: (1) Increase the space between the propagation chains by increasing the distance between the active centers to reduce the probability of entanglement. (2) Control the chain crystallization rate to be higher than the chain growth rate to avoid the entanglement of the uncrystallized polymer chain.

In 1987, Smith et al. discovered that, by employing vanadium chloride (VCl_4_) supported on glass slides as a catalyst under −40 °C, they could obtain UHMWPE, which could be processed under solid conditions [22]. The findings might be explained by the fact that *d*-UHMWPE is produced when polymerization takes place at such low temperatures. The growth chain crystallizes quickly, preventing the formation of entanglements between adjacent chains. However, the catalyst efficiency is relatively low at this temperatures; this method of producing *d*-UHMWPE was not further investigated.

The main challenge for producing UHMWPE lies in how to enhance the chain propagation rate, while mitigating the effects of chain transfer and termination. These issues have been gradually addressed in the studies that have been carried out in recent decades. UHMWPE can be synthesized efficiently through the design of catalysts featuring suitable electronic and steric structures at the metal centers, as well as the screening of appropriate co-catalyst systems and polymerization conditions. Extensive studies on single-site catalysts [23,24,25,26] have facilitated the progression of *d*-UHMWPE. Compared to traditional Z−N catalysts, single-site catalysts can provide polymers with controlled molecular weight and narrow molecular-weight distribution (MWD). By modifying the catalyst structure, the polymerization behavior and the polymer’s structure can be regulated [27,28,29,30,31,32]. In recent years, various highly active and thermally stable single-site catalysts have been reported that are capable of producing UHMWPE with the desired properties (high molecular weight, narrow MWD, low entanglements), which offers the possibility of manufacturing high-modulus, high-strength uniaxial and biaxial tensile tapes and films within a wider temperature window. In addition, compared to the UHMWPE synthesized by Z−N catalysts, the polymers synthesized by single-site catalytic system all exhibit a significantly lower creep rate [33].

To gain a deeper understanding of the entangled state of UHMWPE, various characterization methods, including differential scanning calorimetry (DSC) [34], rheological analysis [35], and solid-state nuclear magnetic resonance (NMR) technology [36], have been adopted. Among them, DSC and rheological analysis are more widely applied. Rheological analysis is performed by using a rotational rheometer, where UHMWPE is subjected to dynamic time scans by maintaining a temperature of 160 °C. During the test, polyethylene chain segments undergo thermal motion at high temperatures, and as the test progresses, chain segments undergo chain entanglement, causing the storage modulus of the melt to increase. Finally, the polyethylene molecular chain segments reach a thermodynamic equilibrium state, and the storage modulus also reaches the equilibrium modulus. There are three main indicators used in rheological analysis to measure the degree of chain entanglement: (1) The ratio of the elastic modulus at a specific time t to the maximum modulus. (2) The time required to reach the thermodynamic equilibrium modulus (*t_m_*) is used as a parameter to measure the degree of chain entanglement. The longer the *t_m_*, the longer it takes for the chain segments to reach thermodynamic equilibrium, indicating a greater degree of chain entanglement in the initial ecological polyethylene. (3) For the initial storage modulus measured at the beginning of the dynamic time scan test, the smaller the initial storage modulus, the larger the average molecular weight between the entanglement points of the initial ecological polyethylene molecular chain, and the smaller the degree of chain entanglement. For DSC, when annealed below the melting point, the molecular chains in the entanglement regions and the amorphous regions detach and melt from the surface of the lamellar crystals. After cooling, the re-arranged melted chain segments will appear in the crystalline regions, and eventually, two melting peaks will be observed in the DSC curve. The lower the degree of entanglement in the amorphous region, the more molecular chains that detach and re-melt from the surface of the lamellar crystals, and a larger area of the low-temperature peak will be presented on the DSC curve. Hence, the degree of chain entanglement of polyethylene molecular chains can be qualitatively evaluated by the ratio of the area of the low-temperature peak to the sum of the areas of the low-temperature and high-temperature peaks.

## 2. Homogeneous Single-Site Catalysis for Synthesizing *d*-UHMWPE

### 2.1. Homogeneous FI Catalyst for Synthesizing d-UHMWPE

In 1998, Fujita et al., from Mitsui Chemicals, synthesized a series of phenoxyimine titanium complexes (FI catalysts) and applied these compounds to olefin polymerization. It was found that the FI catalyst exhibited high catalytic activity for ethylene polymerization [37,38,39]. Some FI catalysts can be used to synthesize *d*-UHWMPE under homogeneous and heterogeneous polymerization conditions. For homogeneous polymerization, the distance between the active sites can be increased by reducing the catalyst concentration and, thus, the distance between growth chains increases. At the same time, lowering the polymerization temperature makes the chain crystallization rate greater than the chain growth rate and inhibits the formation of overlap within the chain.

**Scheme 1:**
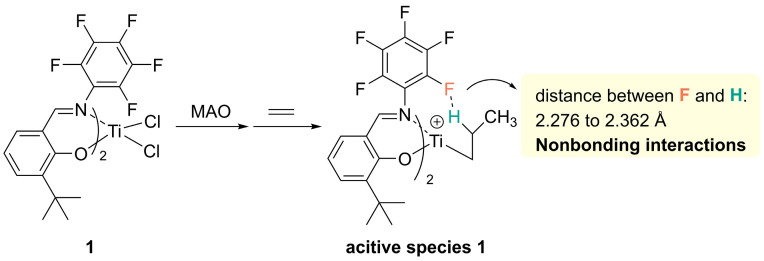
Interaction between ortho-fluorine and *β*-H.

Among the widely used FI catalysts, compound **1** (Scheme 1) has a higher polymerization activity, even at a low temperature. Additionally, the weak interaction between the fluorine atom on the ligand with the *β*-H of the polymer growth chain inhibits *β*-H elimination (Scheme 1) [40]. The DFT calculation on active species **1** showed that the distance between the ortho-F atom and the *β*-H atom is 2.276 to 2.362 Å, which is well within the range of nonbonding interactions. The positively charged *β*-H is stabilized by the electron on the negatively charged fluorine atom, and the electrostatic energy between the F and H atoms is calculated to be approximately −30 kJ mol^−1^. The magnitude of these electrostatic energies is sufficient to reduce the transfer of *β*-H to titanium or reactive monomers. Therefore, compound **1** has been widely used in the preparation of UHMWPE. In 2010, Rastogi’s group reported that compound **1** could produce *d*-UHMWPE with a narrow MWD under the polymerization condition of low temperature and low catalyst concentration [41]. With the use of methylaluminoxane (MAO) as a cocatalyst and 1.3 μmol_cat_/L toluene solution of compound **1**, *d*-UHMWPE with a molecular weight of up to 9.1 × 10^6^ g/mol and polymer dispersity index (PDI) between 1.3 and 2.6 was obtained under room temperature and atmospheric pressure. Key conditions to low entanglement are low temperature and low catalyst concentration, so as to ensure that there is enough distance between the active centers and a greater crystallization rate.

Investigations on polymerization conditions and cocatalysts with FI catalyst to produce *d*-UHMWPE were reported successively (Table 1). In 2014, Rastogi’s group used the FI catalytic system to explore the influence of ethylene pressure on the synthesis of *d*-UHMWPE [34]. When 8.3 μmol_cat_/L toluene solution of compound **1** and Al/Ti ratio of 1200 are used, *d*-UHMWPE can be obtained, with a molecular weight of 2.3 × 10^6^ g/mol at the temperature of 10 °C and the polymerization time of 10 min. The catalytic activity reaches up to 9.7 × 10^6^ g_PE_/(mol_cat_·h·bar). When the ethylene pressure is raised from 1.1 to 2.1 bar (Table 1, entry 1–2), the number average molar mass (*M*_n_) increases from 2.6 to 5.1 × 10^6^ g/mol and does not change considerably if the pressure is further increased to 4.1 bar (Table 1, entry 3). This suggests that, under the conditions used, a *β*-H transfer to the monomer is the most likely chain transfer process. In addition, the mass average molar mass (*M*_w_) rises dramatically when the ethylene pressure rises. This might result from the formation of a secondary catalytic species in the presence of trace amounts of trimethylaluminum (TMA) in MAO. When a lower monomer pressure is used, the mass transfer of ethylene to the active site limits the catalytic activity of secondary species. Thus, by increasing the monomer pressure, and thereby enhancing the monomer dissolution in the reaction medium, a possible mass transfer limitation is likely to be reduced, and the secondary active species may start to contribute more to the overall polymerization. Therefore, the potential mass transfer restriction is likely to be lessened, and the secondary active species may begin to contribute more to the overall polymerization when the monomer pressure is increased. Regarding the entanglement density, it rises in relation to an increase in the ethylene pressure. This could possibly be the result of faster polymerization and rising temperatures near the active site as the monomer concentration rises. The polymerization rate is faster than the crystallization rate.

**Scheme 2:**
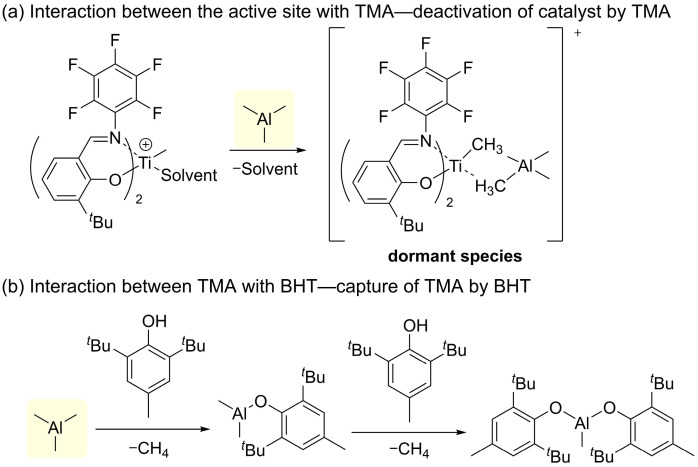
Reactions of TMA with the active site and BHT. (**a**) Interaction between the active site and TMA. (**b**) Interaction between TMA and BHT.

The small amount of TMA that exists in MAO will interact with the active center (Scheme 2a), thus affecting the coordination of ethylene and lowering the catalytic activity. In addition, the chain transfer effect of TMA will also make the resulting polyethylene be of low molecular weight. The addition of the third component, large-hindrance phenol, can effectively capture TMA (Scheme 2b), and the resulting large-hindrance phenoxy aluminum compound has no interaction with the catalyst, and its chain transfer effect is much lower than that of TMA [42,43,44]. Ronca and Rastogi et al. investigated the impact of the third component, 2,6-di-tert-butyl-4-methylphenol (BHT), on the preparation and processing properties of *d*-UHMWPE [45,46]. It was found that, after the addition of BHT in the FI/MAO catalytic system, the polymerization activity could be improved, while the state of low entanglement could be maintained. Under the conditions of 10 °C, 1.1 bar ethylene partial pressure, catalyst concentration of 8.3 μmol_cat_/L toluene solution, and Al/Ti = 1200, the reaction activity was 4.9 × 10^6^ g_PE_/(mol_cat_·h·bar). Disentangled UHMWPE with a molecular weight of 7.2 × 10^6^ g/mol and MWD = 2.7 was obtained (Table 1, entry 4). It was discovered that, in contrast to the other catalytic system, the addition of BHT in this FI/MAO catalytic system produced polymers with lower *M*_w_. According to the study mentioned above [34], instead of being a chain-transfer reagent, TMA reacts with compound **1** and generates a secondary catalyst that is capable of producing chains with higher molecular weights. As for entanglement, the addition of BHT prevents the catalyst from becoming dormant because of TMA, which raises the concentration of active centers and thus leads to a slight increase in the degree of entanglement at the beginning stage of polymerization compared to the catalytic system in the absence of BHT. The difference in the initial entanglement density reduces with the increase in the polymerization time, and it vanishes entirely at the polymerization time of 30 min.

**Table 1:** Compound **1**-catalyzed ethylene polymerization ^a^.

Entry	Pressure (Bar)	Cocatalyst	Al/Ti	Solvent ^b^	Yield (g)	Activity ^c^	*M*_w_ (10^6^ g/mol)	MWD	Ref.
1	1.1	MAO	1200	Toluene	28.4	3.99	9.0	3.4	[45]
2	2.1	MAO	1200	Toluene	51.8	3.96	15.3	3.0	[34]
3	4.1	MAO	1200	Toluene	90.5	3.55	34.0	7.1	[34]
4	1.1	MAO + 1.0 g BHT	1200	Toluene	35.1	4.93	7.2	2.7	[45]
5	1.1	PMAO	2600	Toluene	20.0	2.9	10.7	3.6	[47]
6	1.1	PMAO + 1.2 g BHT	2600	Toluene	26.0	3.90	10.3	3.7	[47]
7	1.1	MMAO12	2600	Toluene	23.0	3.30	7.1	6.7	[47]
8	1.1	MMAO12 + 2.9 g BHT	2600	Toluene	30.0	4.30	7.1	6.7	[47]
9	1.1	MMAO3A	2600	Toluene	3.0	4.00	n.d. ^d^		[47]
10	1.1	MMAO3A + 1.6 g BHT	2600	Toluene	30.0	4.50	8.5	3.0	[47]
11 ^e^	1.1–1.4	MAO	1200	Toluene	23.5	2.20	1.7	3.5	[48]
12 ^f^	1.1–1.4	MAO	1200	T/H 25/70	15.0	1.20	0.9	4.3	[48]
13 ^f^	1.1–1.4	MAO	1200	T/H 50/50	16.0	1.30	1.5	2	[48]
14 ^f^	1.1–1.4	MAO	1200	T/H 75/25	30.2	2.50	2.2	3.2	[48]
15 ^f^	1.1–1.4	MAO	1200	Heptane	12.0	0.97	1.4	2.5	[48]

^a^ Polymerization temperature, 10 °C; Reaction time, 1 h; Solvent amount, 0.75 L; Catalyst concentration, 8.3 μM. ^b^ Toluene/heptane solvent mixtures are expressed as volume%. ^c^ Catalytic activity in 10^6^ g_PE_/(mol_cat_·h·bar). ^d^ Not detected. ^e^ Solvent amount, 0.5–0.75 L; Catalyst concentration, 15 μM. ^f^ Solvent amount, 0.75–1.0 L; Catalyst concentration, 15 μM.

Subsequently, Romano and Ronca et al. explored how various aluminoxane cocatalysts affect *d*-UHMWPE production in the FI catalytic system [47]. Among aluminoxane cocatalysts, unmodified MAO, polymethylaluminoxane-improved performance (PMAO), modified methylaluminoxane type 12 (MMAO12), and modified methylaluminoxane type 3A (MMAO3A) were investigated. The experimental results showed that the cocatalysts play an important role in the activation process. Unmodified MAO with addition of the third component BHT achieved the highest activity, and the polymerization activity was 4.9 × 10^6^ g_PE_/(mol_cat_·h·bar). In order to maintain the same catalyst activity, larger amounts of PMAO, MMAO12 (with and without BHT). and MMAO3A (BHT) were needed than those required for MAO (Table 1, entry 5–10). For all these aluminoxane cocatalysts utilized in this FI catalytic system, the addition of the third component BHT increased the catalytic activity and, especially for MMAO3A, the increase was significant. It has been proposed that the presence of triisobutyl aluminum (TIBA) in MMAO3A will modify the catalyst, and BHT can react with TIBA to protect the catalyst. The entanglement density varies slightly depending on the kind of aluminoxane cocatalyst that is utilized. The FI/MMAO3A catalytic system produces UHMWPE with a relatively higher entanglement density. High-modulus/high-strength aligned fibers in tape geometry are obtained with a tensile strength up to 4.3 N/tex (4.2 GPa) and tensile modulus up to 220 N/tex (210 GPa).

Investigations on the influence of typical polymerization solvents toluene, heptane, and mixed solvents were performed (Table 1, entry 11–15) [48]. The experimental results showed the polymerization activity and *M*_w_ were higher when toluene and heptane were employed as solvents with a specific mixture ratio than that when a single solvent was used. Disentangled UHMWPE was obtained at an activity of 2.5 × 10^6^ g_PE_/(mol_cat_·h·bar) with *M*_w_ of 7.0 × 10^6^ g/mol and the mechanical properties were significantly improved. According to the experimental results, a larger dielectric constant of toluene can promote the ionic dissociation of the catalyst/cocatalyst ionic pair, bringing ethylene closer to the active center. In addition, the solubility of ethylene in heptane is higher than that in toluene. Consequently, the combination of toluene and heptane has a synergistic effect that is more advantageous than that of a single solvent due to the enhancement in the ethylene concentration, which prevents the “starvation” of the active center.

These studies offer valuable strategies for the synthesis, characterization, and processing of nascent *d*-UHMWPE. As demonstrated by the use of the FI catalyst/MAO homogeneous polymerization system to obtain *d*-UHMWPE under low temperature and high dilution, the key factors to low entanglement are to maintain a sufficient distance between the active sites and ensure that the crystallization rate is greater than the chain propagation rate. Low temperature and high dilution are the necessary conditions to obtain *d*-UHMWPE for a homogeneous FI catalyst. The main advantage of using the FI catalyst is its high catalytic activity, even at the low temperature of 10 °C. The catalytic activity can still be kept above 10^6^ g_PE_/(mol_cat_·h·bar) and even become close to 10^7^ g_PE_/(mol_cat_·h·bar) after condition optimization. Nevertheless, 10 °C is far too low from the actual industrial production temperature of polyolefins, and the large amount of solution used makes it not feasible for industrial production.

### 2.2. Other Types of Homogeneous Single-Site Catalysts for Synthesizing d-UHMWPE

In recent years, various class of single-site catalysts, including titanium- and chromium-based half-metallocene catalysts and post-metallocene catalysts containing titanium and vanadium centers, have also been utilized in the preparation of *d*-UHMWPE. The majority of these catalysts are homogeneous.

**Figure 2:**
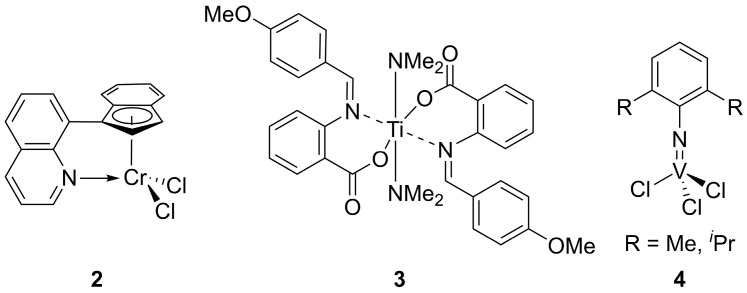
Other types of homogeneous catalysts.

Romano and Rastogi et al. found that the entanglement density in the amorphous region of semi-crystalline UHMWPE could be adjusted under appropriate conditions by using chromium-based single-site catalyst (Figure 2, compound **2**) to obtain *d*-UHMWPE [49]. Compound **2**/MAO system can be modified by adding BHT to increase the molecular weight of the resulting PE to an ultra-high molecular weight range while preserving a low degree of entanglement. Under the condition of 10 °C and an ethylene partial pressure of 1.1 bar, with catalyst concentration of 6–8 μmol_cat_/L toluene solution, the polymerization activity can reach up to 5.4 × 10^6^ g_PE_/(mol_cat_·h·bar). Uniaxially drawn tapes with a tensile strength and modulus greater than 3.5 N/tex and 200 N/tex, respectively, were obtained through solid-state processing below the equilibrium melting temperature.

In 2023, Chikkali’s group reported ethylene polymerization catalyzed by amido Ti-iminocarboxylate complex (Figure 2, compound **3**) [50]. It was found that *d*-UHMWPE with a molecular weight of 2.5 × 10^6^ g/mol and a crystallization χ (DSC) of 96% was produced by using modified methylaluminoxane (MMAO) as the cocatalyst under 4 bar ethylene pressure, polymerization temperature of 35 °C, and polymerization time of 30 min.

In early 2000, Nomura et al. reported a series of complexes based on (imido)vanadium precursors and demonstrated that these complexes were capable of producing UHMWPE [51,52]. Due to its easy synthesis and high catalytic efficiency, Blom and Romano et al. used the (imido)vanadium(V) trichloro complexes (Figure 2, compound **4**) to explore the preparation of *d*-UHMWPE [53]. Complexes **4** could be easily synthesized by a one-step reaction from aryl isocyanate with vanadium oxychloride (VOCl_3_). Disentangled UHMWPE can be obtained with a catalytic activity up to 3.4 × 10^5^ g_PE_/(mol_cat_·h·bar) in the presence of MAO and 12 μmol/L catalyst toluene solution at 10 °C. The peak melting temperatures of polymers range from 130 to 140 °C and the crystallinity is about 62 to 66%.

**Scheme 3:**
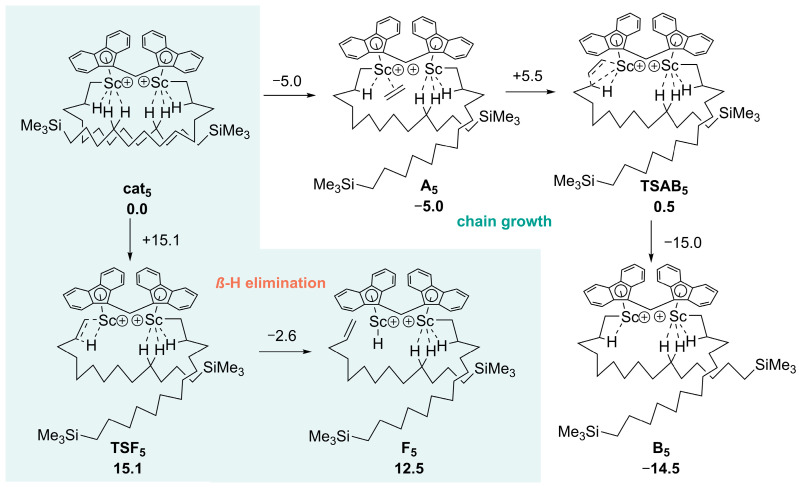
Energy profiles for the chain growth and *β*-H elimination of ethylene polymerization catalyzed by **5** from the DFT simulation (ΔG, kcal mol^−1^).

In 2023, Cui and Li et al. demonstrated that a rare-earth scandium compound (Table 2, compound **5**) could catalyze the formation of *d*-UHMWPE through binuclear synergic effect and agostic interaction [54]. The polymerization results show that, in comparison to the mononuclear Sc compound Sc1, methylene-bridged binuclear Sc compound **5** has a higher catalytic activity and may produce polymers with a higher molecular weight (Table 2, entry 1, 3). Moreover, the compound C2-Sc2, which the methylene linker of compound **5** changes into a flexible ethylene bridge, has no effective synergistic impact and two active species are interfered (Table 2, entry 2). The DFT calculation simulates the olefin polymerization processes catalyzed by **5** (Scheme 3) and its mononuclear analogue, respectively. The H atom on the polymer chain dissociates from the Sc center during the ethylene coordination process when binuclear Sc compounds are used, releasing 5 kcal mol^−1^ energy. As a result, chain growth is easier since the energy barrier needed for ethylene coordination and insertion is lower than when employing mononuclear Sc as a catalyst (0.5 kcal mol^−1^ vs. 10.1 kcal mol^−1^). Moreover, when using binuclear Sc compounds, the energy barrier difference between chain growth and *β*-H elimination is higher than using mononuclear Sc compounds (9.6 kcal mol^−1^ vs. 5.1 kcal mol^−1^). Thus, a polymer with a higher molecular weight was obtained (Table 2, entry 1–2). Binuclear synergies and interaction between active centers with growth chains contribute to the low entanglement property of polymers. During the early stages of polymerization, agostic interaction between the metal centers Sc and H on the polymer chain makes polymer chains growing from two active sites that are arranged into conformationally ordered segments. When the ordered segments exceed a certain size, they spontaneously aggregate into crystalline nuclei, triggering crystallization to form disentangled structures even at a relatively high temperature. Disentangled UHMWPE can be obtained at wide ranges of temperature (25–120 °C) and ethylene pressure (2–13 bar), with polymerization activity up to 1.8 × 10^6^ g_PE_/(mol_cat_·h·bar) and molecular weight up to 2.8 × 10^6^ g/mol. The boundaries of homogeneous polymerization at a low polymerization temperature and a low ethylene pressure under the highly diluted catalyst concentration used to produce *d*-UHMWPE were pushed. These *d*-UHMWPE materials are easy to be processed in solid state at 130 °C, and their tensile strength and modulus reach 149.2 MPa and 1.5 GPa, respectively.

The above results demonstrate that the majority of other homogeneous catalyst types are similar to FI catalysts in that a low temperature and low catalyst concentration are necessary to achieve low entanglement. However, for the binuclear scandium catalyst, a low temperature is no longer a necessary condition to obtain *d*-UHMWPE through binuclear synergic effect and agostic interaction. The novelty to us is the innovation of the catalyst structure, which can enable a wider temperature and pressure window through the interaction between the catalyst and polymer chain.

## 3. Heterogeneous Single-Site Catalysis for Synthesizing *d*-UHMWPE

Disentangled UHMWPE can also be prepared by heterogeneous polymerization. The heterogenization of single-site catalysts can promote their thermal stability and improve their catalytic properties while maintaining the single-sites characteristics of catalysts. In addition, this heterogenization enables the control of the polyolefin morphology to reduce reactor fouling and match the requirements of the current polyolefin production process. Additionally, it has been demonstrated that a significant decrease in the proportion of MAO may be achieved in comparison to homogeneous conditions to maximize the catalyst’s performance [55].

**Figure 3:**
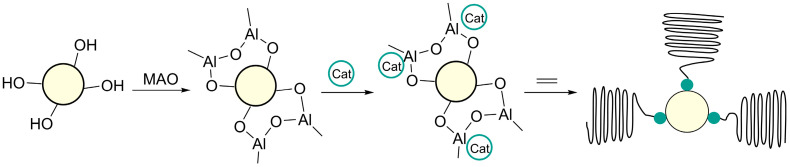
Catalyst-loading process and the diagram of heterogeneous ethylene polymerization.

In 2012, Ronca and Rastogi et al. impregnated MAO on high-surface area of nanoparticles, such as ZrO_2_, TiO_2_, hydroxyapatite (Hap), and carbon nanotubes (CNTs) [56]. Then, the FI catalyst compound **1** was added to obtain heterogeneous single-site catalysts (Figure 3). The synthesis of disentangled polymers can be ensured when the active site is widely distributed on the nano-surface. At room temperature, heptane was used as a solvent and TIBA as a scavenger, with a 1.1 bar partial pressure of ethylene, resulting in UHMWPE. Rheology showed that the obtained polymer had a low initial modulus and the modulus buildup with time, indicating a low entanglement state, and high-performance composite tapes and films were prepared by solid-state processing. The polymerization results demonstrated that TiO_2_ and CNT, acting as carriers, have a higher catalytic polymerization activity (Table 3). This provides an approach toward the utilization of single-site catalytic systems in slurry and gas phases, with a marked reduction in the use of MAO and reactor fouling.

**Figure 4:**
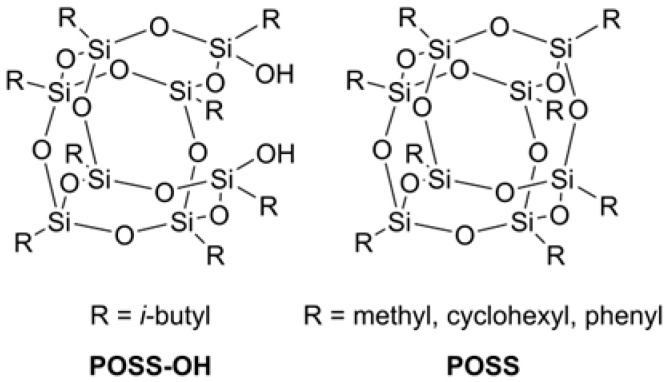
Structure of POSS.

In 2014, Li’s group synthesized disentangled UHMWPE/polyhedral oligomeric silsesquioxane (POSS) nanocomposites by loading the FI catalyst on disilanolisobutyl POSS with a hydroxyl group (Figure 4, POSS-OH) [57]. It was suggested that there might be a strong interaction between the POSS particles and the growing polymer chains. The POSS particles in the solution are preferably adsorbed on the surface of the nascent polymers, which could have an impact on the entanglements of polymers. Furthermore, POSS particles may act as nucleating agents, thus enhancing the crystalline behaviors of the nascent UHMWPE. Subsequently, various types of polyhedral oligomeric silsesquioxane (methyl-POSS, cyclohexyl-POSS, and phenyl-POSS) combined with the FI catalyst compound **1** were explored [58]. The results showed that all the nanocomposites had a lower initial storage modulus, which reflected the low entanglement structure. Among these obtained nascent UHMWPE nanocomposites, entanglement increases in the order of UHMWPE/POSS-C_6_H_12_, UHMWPE/POSS-C_6_H_6_, and UHMWPE/POSS-CH_3_.

By utilizing MAO as a linker, the FI catalyst compound **1** was effectively immobilized on the graphite oxide (GO) surface [59]. A small number of active centers spread across the GO surface to keep the propagation chains apart and a comparatively low polymerization temperature of 20 °C was employed to ensure low entanglement. The UHMWPE/GO nanocomposites were obtained with notable draw ratio, demonstrating their low entanglement density.

In 2022, Romano and Rastogi et al. explored the preparation of *d*-UHMWPE with compound **1** as a catalyst and magnesium chloride/ethylaluminum 2-ethyl-1-hexanolate (MgCl_x_/Et_n_Al_y_(2-ethyl-1-hexanolate)_z_) as the support and activator [60]. MgCl_x_/Et_n_Al_y_(2-ethyl-1-hexanolate)_z_ was formed in situ at 50 °C from triethylaluminum and MgCl_2_/2-ethyl-1-hexanol adduct, and then, the polymerization was carried out at 4 bar of ethylene partial pressure at 40 °C. The synthesized activator/carrier was nanoscale-sized particles with a homogeneous chemical composition distribution and thus lead to a decreased amount of active sites on which they were supported. As a result, chain overlap was avoided to a great extent. Under optimized conditions, the polymerization activity reached up to 1.6 × 10^6^ g_PE_/(mol_cat_·h·bar) and the generated *d*-UHMWPE had a molecular weight in the range of 4.6–14.7 × 10^6^ g/mol, which is suitable for solid-state solvent-free uniaxial and biaxial processing. The ultimate tensile strength reached 4.0 N/tex and tensile modulus was greater than 200 N/tex.

**Figure 5:**
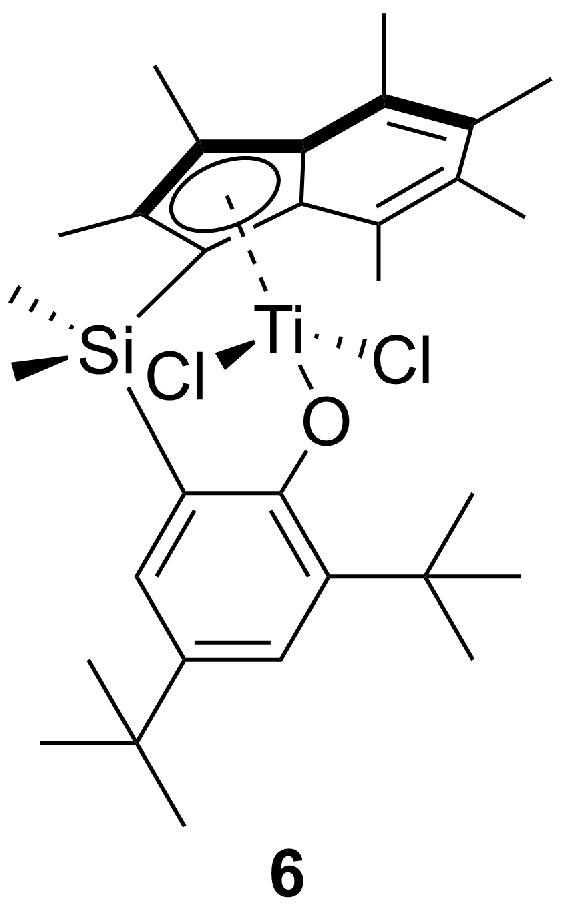
Compound **6**.

In 2021, O’Hare et al. reported a novel permethylindenyl-phenoxide (PHENI*) metallocene titanium complex (Figure 5, compound **6**) and supported it on an inorganic solid carrier [61]. The activity of slurry-phase ethylene polymerization reached 3.7 × 10^6^ g_PE_/(mol_cat_·h·bar) at 60 °C when the carrier was solid MAO. Polyethylene with disentangled characteristics was obtained, and the *M*_w_ was 3.4 × 10^6^ g/mol.

A self-immobilized catalyst usually has unsaturated groups, which will copolymerize with the polymer. It is an easy way to heterogenize single-site catalysts. During the polymerization process, a catalyst supported on polymers occurs so that homogeneous catalytic system gradually changes to heterogeneous. Polyolefins with improved morphology and increased molecular weight can be obtained, and reactor fouling can be reduced [62,63]. Several types of single-site catalysts, such as (half-)metallocene, post-metallocene, and late-transition-metal catalysts, have been modified into self-immobilized polyolefin catalysts [64]. Among them, FI catalysts with diallylamino or alkenyloxy groups are being researched extensively [65,66,67,68]. It was found that *d*-UHMWPE could be obtained by ethylene polymerization with a self-immobilized catalyst at a higher temperatures. A low temperature is not essential for rapid crystallization when heterogeneous nuclei exist near the growing polymer chain. Apparently, the carrier polyethylene crystal for immobilizing the catalyst is a heterogeneous nuclei.

**Figure 6:**
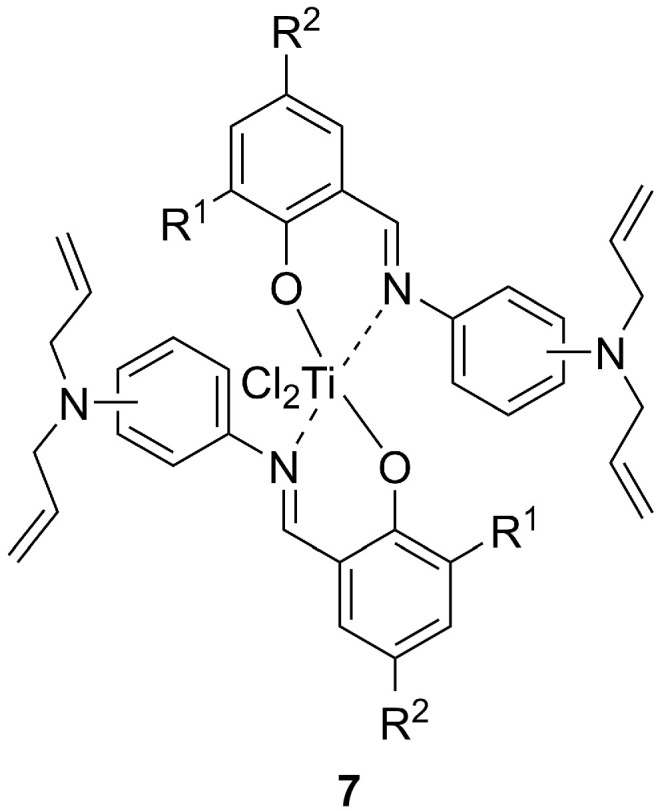
Self-immobilized FI catalyst.

In 2020, Oleyinik et al. synthesized a series of self-immobilized FI catalysts containing a diallylamino group (Figure 6, compound **7**) and investigated the influence of the catalyst structure and polymerization conditions on polymerization kinetics, catalytic activity, molecular weight, microstructure, and morphology in detail [69]. The structure–activity relationship study showed that the structure of the substituent R^1^, R^2^, and substituted position of diallylamino group have different impacts on the activity, molecular weight, melting temperature, and crystallinity (Table 4). When a catalyst with R^1^ = CMe_2_(Ph), R^2^ = H, and N(CH_2_CH=CH_2_)_2_ at the meta-position is used, the polymerization has the highest activity of 1.4 × 10^6^ g_PE_/(mol_cat_·h·bar) (Table 4, entry 5). When R^2^ is replaced by Me, the molecular weight of the polymer reaches up to 4.1 × 10^6^ g/mol (Table 4, entry 6). In terms of mechanism, after the pre-catalyst self-immobilizes on the carrier polyethylene macromolecule, the active center is close to the surface of the carrier crystal suitable for nucleation. Because the contact surface has the same exact lattice matching, the molecular chains grown from the supported active center do not have a nucleation barrier and can rapidly crystallize on the surface of the carrier to form *d*-UHMWPE even at high temperatures. When the catalyst concentration was 24 μmol/L and the reaction temperature was in the range from 30 to 60 °C, the molecular weight of UHMWPE was 1.70–4.40 × 10^6^ g/mol with low entanglement.

**Figure 7:**
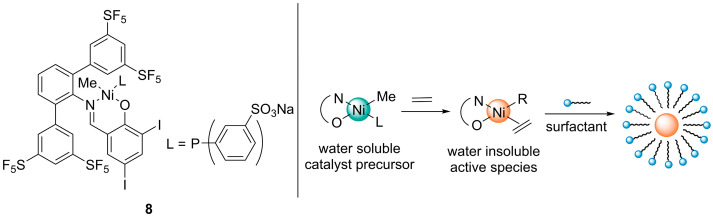
Water-soluble Ni(II) complex **8** and the mechanism of aqueous-phase polymerization.

The compartmentalization of active centers is an effective means to obtain *d*-UHMWPE. Most metal catalysts used in olefin polymerization, such as Ti-, Zr-, V-, and Cr-centered catalysts, are water-sensitive and become inactive when exposed to water. Late-transition metals, however, have a strong water tolerance and comparatively low oxygen philicity. Mecking’s group developed a class of Ni (II) compounds that are water-tolerant, and the electronic effects and steric hindrance of the ligand structure can avoid the shortcomings of chain transfer by the *β*-H elimination of Ni catalysts during ethylene polymerization [70,71,72]. The water-soluble Ni(II) complex **8** (Figure 7) containing a highly electron-withdrawing substituent pentafluorosulfanyl (SF_5_) group can produce *d*-UHMWPE under aqueous conditions [73]. The pentafluorosulfanyl group is more electron-withdrawing than the commonly used trifluoromethyl (CF_3_) group, and thus, the utilization of a catalyst with the SF_5_ group can significantly reduce the undesired *β*-H elimination during ethylene polymerization. Under the conditions of 40 bar ethylene partial pressure and temperature of 15 °C, adding an appropriate amount of surfactant sodium dodecyl sulfate (SDS) and pH regulator CsOH·H_2_O, *d*-UHMWPE with a particle size of 25–27 nm can be obtained. The polymerization activity reached up to 4.0 × 10^4^ g_PE_/(mol_cat_·h·bar), and the molecular weight reached a maximum of 1.4 × 10^6^ g/mol. Previous studies have shown that water-soluble catalyst precursors will first become lipophile active species that are insoluble in water during the catalytic polymerization of olefin, and the active center is surrounded by a large number of surfactants present in the aqueous solution to form a segmented independent space, which can then form a low-entanglement polymer [70]. In recent years, there has been a growing awareness of environmental protection, and aqueous polymerization, an environmentally friendly method, has gained increased attention. The advantages of aqueous polymerization include efficient heat transfer, non-toxic and non-flammable solvents, and no need for water-sensitive cocatalysts [74].

**Figure 8:**
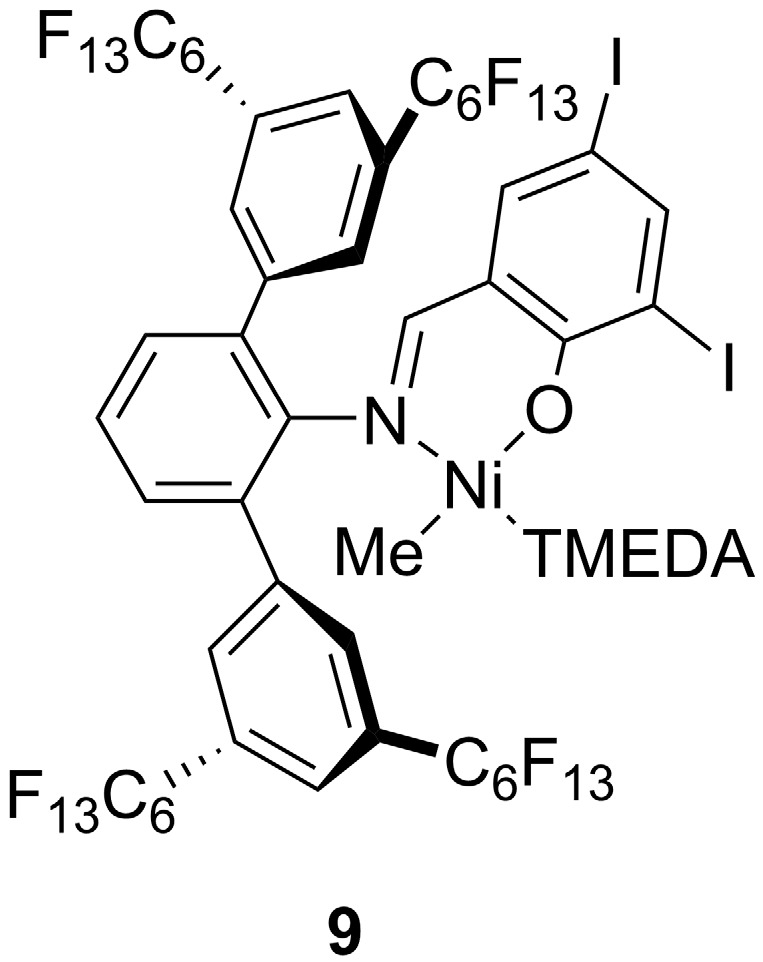
Lipophilic Ni(II) complexe **9**.

In microemulsion polymerization, each droplet can be an ideal single separation system to produce low-entanglement polyethylene. In 2021, Mecking’s group obtained *d*-UHMWPE by the microemulsion polymerization of lipophilic Ni(II) catalyst **9** (Figure 8) [75]. Compound **9** was synthesized in situ by *N*,*N*,*N*′,*N*′-tetramethylethylenediamine (TMEDA) dimethyl nickel and salicylaldimine. It was then dissolved in a small volume of water-immiscible organic solvent and dispersed via emulsification by ultrasonication in a large volume of surfactant aqueous solution. By polymerization at 40 bar ethylene partial pressure and 15 °C for 30 min, the *d*-UHMWPE with a molecular weight of 2.1 × 10^6^ g/mol, MWD of 2.3, and particle size of 402 nm was produced. The catalytic activity was 2.1 × 10^4^ g_PE_/(mol_cat_·h·bar).

## 4. Conclusions

In summary, *d*-UHMWPE can be obtained with both homogeneous and heterogeneous single-site catalysts. The studies revealed how polymerization conditions contribute to entanglement density, morphology, and melting behavior of nascent *d*-UHMWPE. Studies demonstrated that a large distance between active sites and a crystallization rate greater than the chain growth rate are two key factors to low entanglement. A low polymerization temperature and high catalyst solution dilution are necessary for the majority of homogenous single-site catalysts in order to fulfil the two requirements. These catalysts must be able to exhibit a certain degree of catalytic activity at low temperatures. Additionally, the synthesis of *d*-UHMWPE can be achieved by compartmentalizing the active sites using microemulsion and aqueous polymerization. At present, heterogeneous single-site catalysts are closer to the industrial production of *d*-UHMWPE compared to homogenous catalysts. Among these, using self-immobilized catalysts accelerates the crystallization rate of growing chains, ensuring that, even at higher temperatures, the rate of crystallization will still be higher than the rate of chain growth. How to reduce entanglement more efficiently while maintaining a high molecular weight and narrow molecular-weight distribution remains a crucial issue for future research. Single-site catalysts will continue to be an appealing choice for the synthesis of *d*-UHMWPE in the long term. As for processing approaches, the melt processing of *d*-UHMWPE is comparatively undeveloped, and it is desperately needed that it is advanced in the future. As the demand for lightweight yet durable materials continues to escalate, particularly within sectors centered on enhancing energy efficiency and minimizing emissions, the exploration of polymer composites and blends integrating *d*-UHMWPE is anticipated to gather pace. Current research indicates that *d*-UHMWPE may find extensive use in high-value-added fields such as total knee/hip/ankle joint replacements, photochromic devices, and electronic devices in the near future. Further extending the application of *d*-UHMWPE should be an essential subject of research.

## Figures and Tables

**Table 2:** Ethylene polymerization catalyzed by scandium compounds ^a^.

Entry	Pre-Catalyst	Temp. (°C)	Pressure (Bar)	Time (Min)	Yield (g)	Activity ^b^	*M*_w_ (10^6^ g/mol)	MWD
1	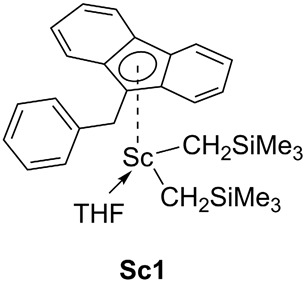	80	4	1	28.4	0.45	0.4	2.6
2	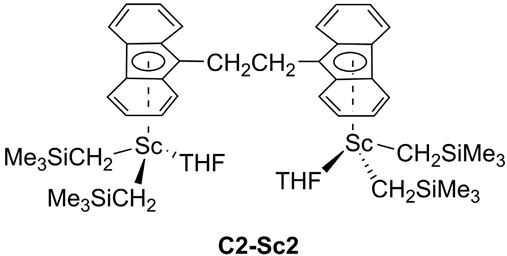	80	4	1	51.8	0.38	0.2	6.0
3	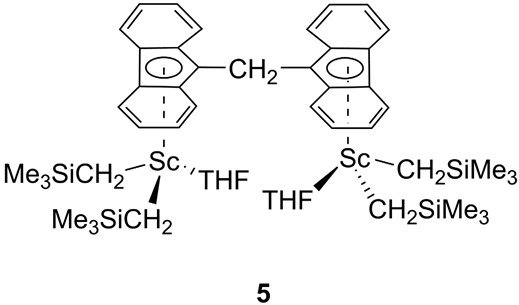	80	4	1	90.5	1.42	1.2	2.0
4		20	4	1	35.1	1.80	2.8	1.9
5		60	4	1	20.0	1.50	1.8	1.7
6		120	4	1	26.0	5.40	1.2	1.6
7		80	2	1	23.0	1.80	1.3	2.9
8		80	6	1	30.0	1.00	1.9	2.3
9 ^c^		25	13	10	12.0	1.82	1.5	2.6

^a^ [Sc], 20 μmol; [Sc]/[Ph_3_C][B(C_6_F_5_)_4_]/Al*^i^*Bu_3_ = 1:1:5 (mol/mol/mol); Toluene, 0.2 L. ^b^ Catalytic activity in 10^6^ g_PE_/(mol_cat_·h·bar). ^c^ Hexane, 1.5 L; [Sc]/[Ph_3_C][B(C_6_F_5_)_4_]/Al*^i^*Bu_3_ = 1:1:500 (mol/mol/mol).

**Table 3:** FI heterogeneous ethylene polymerization supported by different nanoparticles ^a^.

Entry	Support Type	FI Catalyst (μM)	Al_MAO_/Ti	Al_TIBA_ (mmol)	Heptane (L)	Yield (g)	Activity ^b^
1	TiO_2_	11	73	2	0.2	7.8	6.82
2	ZrO_2_	11	68	2	0.2	3.0	2.45
3	Hap	8.8	20	2	0.2	3.0	3.03
4	CNT	30	133	25	5.0	22.0	6.24

^a^ Polymerization temperature, 25 °C; Reaction time, 1 h; Reaction pressure, 1.1 bar. ^b^ Catalytic activity in 10^5^ g_PE_/(mol_cat_·h·bar).

**Table 4:** Compound **7**-catalyzed ethylene polymerization ^a^.

Entry	Pre-Catalyst			Yield (g)	Activity ^b^	*M*_w_ (10^6^ g/mol)	Tm		*Χ* (%)	
	R^1^	R^2^	N(Allyl)_2_				1°	2°	1°	2°
1	*t*-Bu	H	m	3.3	6.85	1.0	140.4	136.0	85.6	53.0
2	*t*-Bu	Me	m	2.7	5.65	1.3	141.0	135.4	80.8	50.0
3	*t*-Bu	OMe	m	5.2	10.80	1.4	137.5	136.0	83.3	60.6
4	*t*-Bu	*t*-Bu	m	2.4	5.06	2.3	143.7	136.5	83.5	52.1
5	CMe_2_(Ph)	H	m	6.7	14.00	3.0	140.1	134.9	83.1	49.3
6	CMe_2_(Ph)	Me	m	4.1	8.60	4.1	140.8	136.2	84.6	47.3
7	CMe_2_(Ph)	OMe	m	2.5	5.17	2.4	139.8	135.9	85.3	53.8
8	CMe_2_(Ph)	*t*-Bu	m	5.5	11.50	2.3	140.5	136.1	84.6	48.7
9	*t*-Bu	H	o	5.2	10.90	1.1	137.7	136.2	82.8	58.1
10	*t*-Bu	Me	o	3.0	6.17	1.2	137.6, 140.7	136.2	83.9	56.6
11	*t*-Bu	OMe	o	4.5	9.35	0.9	137.0, 140.4	132.8	83.1	53.5
12	*t*-Bu	*t*-Bu	o	4.4	9.27	1.0	139.6, 142.8	137.0	79.3	51.6
13	CMe_2_(Ph)	H	o	3.4	7.04	1.5	137.5	136.6	82.9	62.6
14	CMe_2_(Ph)	Me	o	2.1	4.29	1.0	137.6	136.9	78.7	58.2
15	CMe_2_(Ph)	OMe	o	2.4	5.08	0.7	136.5	129.1	78.2	52.7
16	CMe_2_(Ph)	*t*-Bu	o	2.8	5.85	1.4	138.2	133.3	78.2	53.9

^a^ Conditions: 1.2 × 10^−6^ mol of pre-catalyst, MAO as cocatalyst (Al/Ti = 500), 40 °C, 1 h, 4 bar of ethylene, and toluene (50 mL) as solvent. ^b^ Catalytic activity in 10^5^ g_PE_/(mol_cat_·h·bar).
